# In Situ Tensile Deformation and Mechanical Properties of α Platelets TC21 Alloy

**DOI:** 10.3390/ma15113869

**Published:** 2022-05-28

**Authors:** Chunlin Yang, Song Zhang, Meigui Ou

**Affiliations:** 1 College of Chemistry and Materials engineering, Guiyang University, Guiyang 550005, China; ycl770309@163.com; 2 College of Materials and Metallurgy, Guizhou University, Guiyang 550025, China; justin-song@foxmail.com; 3 Key Laboratory for Material Structure and Strength of Guizhou Province, Guiyang 550025, China

**Keywords:** TC21 alloy, α platelets, mechanical properties, in situ tensile deformation

## Abstract

The present study was focused on the relationship between an α platelet microstructure and the properties of TC21 alloy, and the tensile deformation process was revealed by in situ observation. To obtain the α platelet microstructures, the samples were administered a solution treatment (1000 °C for 15 min) and then cooled to room temperature by different cooling methods (furnace cooling (FC), open-door furnace cooling (OFC), air cooling (AC), and water quench (WQ), corresponding to an increased cooling rate). It is found that α platelets become thinner and colonies become narrower with the increase in cooling rate. The formation of the platelet microstructure is based on the preferred Burgers orientation relationship of {110}_β_//{0001}_α_ and <111>_β_//<11$\bar{2}$0>_α_. The α platelets orientation changes with the cooling rate. These differences in α platelets thickness and orientation result in the excellent ductility of the sample with thick platelets and the high strength of the samples with thin platelets. During the in situ tensile deformation process, the crack propagation path is deflected in the presence of grain boundaries, α platelets, and α colonies with different orientations. The fracture of the sample with thick α platelets shows better ductility compared to those with thin α platelets.

## 1. Introduction

Since the 1940s, titanium (Ti) alloys such as TC4 (Ti-6Al-4V) and TC21 (Ti-6Al-2Zr-2Sn-3Mo-1.5Cr-2Nb) have been produced and widely applied as critical structural components of aircraft due to their low density, high strength, and toughness [1,2]. Ti alloys with a full-lamellar microstructure have excellent fracture toughness, which can prevent the rapid propagation of cracks once they occur. Therefore, the deformation and fracturing of Ti alloys with platelets have garnered significant research attention [3].

A large number of studies focusing on the deformation behavior of Ti alloys have discussed the machinability and microstructural transformations at elevated temperatures [4,5,6,7,8]. The α platelets, derived from forging or pretreatment at β region, play an important role in grain refinement when the sample was forged in the α+β region. Therefore, several researchers studied the relationship between α platelets structures and their mechanical performance [3,9,10,11]. The study based on Ti-6Al-4V alloy showed that with the increase in cooling rate, the α platelets became smaller and finer, the tensile strength increased, and the ductility decreased [3]. Filip et al. elaborated that the length and thickness of α phase decreased and the yield stress increased with an increasing cooling rate [9]. Yadav stated that a full platelet structure has excellent fracture toughness and crack propagation resistance [10,11]. In recent years, in situ tensile experiments have been carried out to observe the deformation behavior of Ti alloys [12,13,14]. It has been reported that the slip bands, appearing in stress concentration zones, accumulated to form shear bands and micro-cracks were induced at the interface of α platelets and shear bands, leading to crack propagation and fracture [9,10]. However, continuous slips in the coarse α phase delayed the crack propagation and led to excellent ductility [14,15]. Castany et al. [16] indicated that interfaces improved the alloy strength by suppressing the dislocation movement and facilitating plastic deformation. Since the existence of Burgers Orientation Relationship (BOR) in Ti alloys (i.e., ${\{110\}}_{\beta }$//${\{0001\}}_{\alpha }$, ${\langle 111\rangle }_{\beta }//{\langle 11\bar{2}0\rangle }_{\alpha }$), the dislocation slips easily through the β platelets in a straight line or a deflection of 10.5° during plastic deformation, which results in the decreased strength of the platelet structure [17]. On the contrary, the platelet structure obtained by cooling from the β region often had high toughness and resistance to crack propagation, and its properties were sensitive to the thickness of α platelets [18].

Although the previous research has been elaborate, the effect of the microstructure morphology on the properties, the relationship between full-lamellar microstructure, and the mechanical properties of TC21 alloys were rarely studied. In addition, the slip transfer between α or β platelets and colonies and the mechanism of crack propagation are also inexplicit. Therefore, the purpose of this paper is to study the influence of lamellar thickness obtained at different cooling rates on the mechanical properties of the titanium alloy and its tensile deformation mechanism. The current work provides novel insights into the damage mechanism of the TC21 alloy and a theoretical basis for the determination of the optimal microstructure so as to obtain a TC21 alloy with excellent mechanical properties.

## 2. Materials and Methods

**Figure 1:**
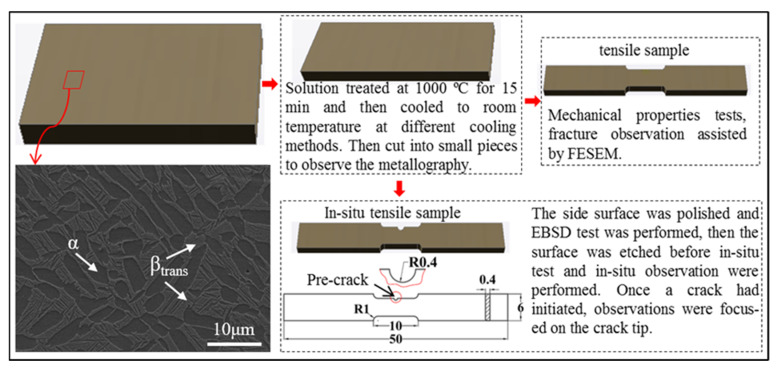
Schematic preparation process of the tensile specimen and original microstructure of TC21 alloy (lower left) (units of tensile sample: mm).

As-received TC21 alloy (Ti-6.47Al-2.28Zr-2.18Sn-3.23Mo-1.51Cr-2.11Nb-0.11Si) was a circular forged bar with a β-transformation temperature (T_β_) of 975 ± 5 °C. The preparation process of specimens is schematically shown in Figure 1. The specimens were put in the furnace and the temperature increased to 1000 °C, maintained for 15 min, and then cooled to room temperature at different cooling methods: furnace cooling (FC, corresponding to an approximate cooling rate of 0.5 °C·s^−1^), open-door furnace cooling (OFC, 2 °C·s^−1^), air cooling (AC, 15 °C·s^−1^), and water quench (WQ, 140 °C·s^−1^). As reported earlier, different thicknesses of α platelets can be obtained by using different cooling rates [19].

The metallographic specimens were ground by SiC paper and polished, then etched in the mixed solution (HF:HNO_3_:H_2_O = 1:3:7) for 1–2 s. The morphologies of the microstructure and fracture were collected by a field-emission scanning electron microscope (FESEM, ZEISS SUPRA 40, Carl Zeiss AG, Oberkochen, Germany) and an atomic force microscope (AFM, Bruker Dimension Icon, Bruker Corporation, Billerica, MA, USA). The specimens for the electron back scattered diffraction (EBSD) test were polished using a vibratory finishing machine (Buehler Vibromet 2, Illinois Tool Works Inc; ITW, Glenview, IL, USA). The EBSD patterns were obtained by an FESEM equipped with an EBSD detector and analyzed by Channel 5 software. The tensile specimens with a gauge length of 30 mm and a diameter of 6 mm were prepared by electrical discharge machining (EDM), and the tensile tests were performed in the MTS-GWT2105 machine (MTS System Corporation, Marblehead, MA, USA).

The specimens for the in situ tensile tests were processed into the desired shape and then performed standard metallographic procedures, as shown in Figure 1. The circular notch was set as the pre-crack on one side of the sample to facilitate the observation of the crack’s initiation and propagation. Owing to the stress concentration at the notch during the tensile process, the micro-cracks would be produced near the notch. The in situ tensile test was carried out using a tensile tester placed inside FESEM under a maximum loading force of 5000N. The tensile strain rate was controlled by the displacement of 0.5 μm·s-1. The SEM images were obtained during the intervals of loading, and the related data (lamellar α thickness, colony size, and width of grain boundary (GB)) were calibrated by the software Image pro plus 6.0 (Media Cybernetics Corporation, Rockville, MD, USA).

## 3. Results and Discussion

### 3.1. Microstructure

**Figure 2:**
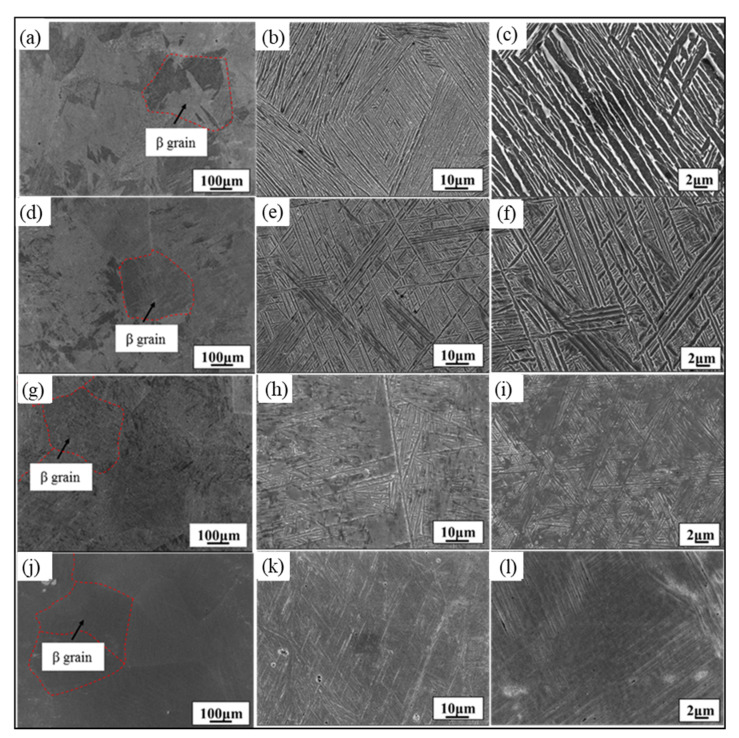
Microstructure of different cooling methods: (**a**–**c**) FC; (**d**–**f**) OFC; (**g**–**i**) AC; and (**j**–**l**) WQ.

The microstructures of the TC21 alloy under different cooling methods are shown in Figure 2. The specimen after heat treatment presents a multi-level microstructure with primary α and intercrystalline β. The related parameters of the microstructure are listed in Table 1. There are large numbers of α platelets in the microstructure, and the α platelets in the same orientation form an α colony in Figure 2a–f.

The cooling methods (rates) play a key role in the formation of α platelets, and the change of original β grain size is slight at relatively fast cooling methods (e.g., OFC, AC, and WQ, from 250.31 to 238.15 μm). The other parameters (thickness of α platelet, α colony, GB, and α+β platelets) are relatively high at slow cooling rates (FC) and slowly vary at the relatively fast cooling rates. The appearance of martensitic microstructure in case of WQ demonstrates that the previously α formed platelets will reduce the available volume for new nucleation and cause a smaller size of α platelet in Figure 2j–l. In this case, the grain boundary of α phase is no longer obvious, and the α+β platelets are difficult to observe and measure.

**Figure 3:**
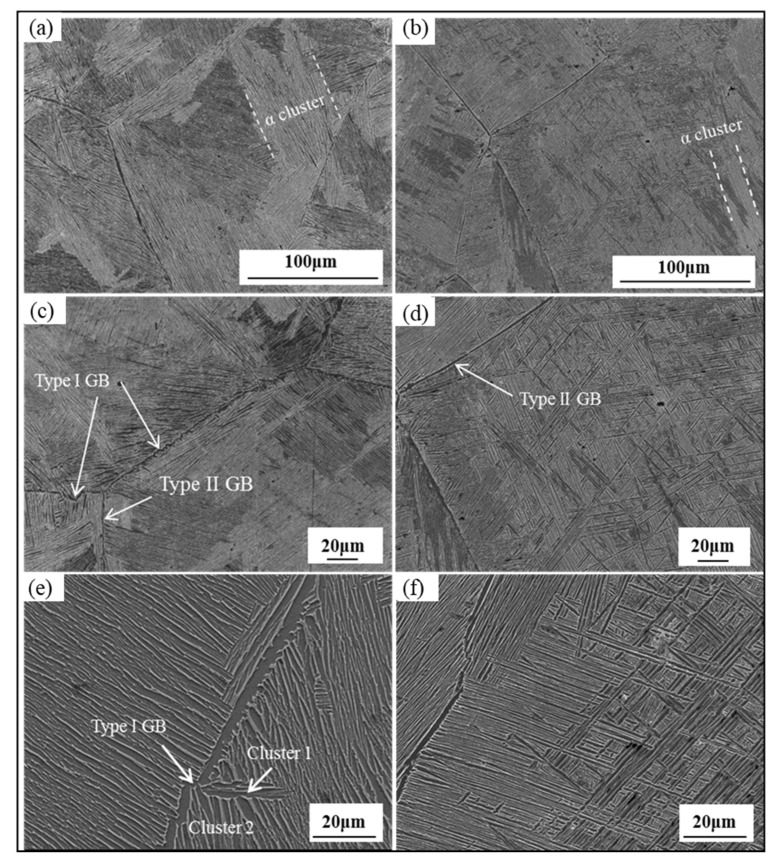
Microstructure of FC (**a**,**c**,**e**) and OFC (**b**,**d**,**f**).

Owing to the low supercooling degree and high GB energy, the α phase easily nucleates at the GB at low cooling rates, and then gradually grows into the entire grain to form thick platelets, as seen from Figure 3a,c,e. Two types of GB in FC specimen are observed: the first type of GB (Type I) has a zigzag appearance in Figure 3c and results from separate heterogeneous nucleation and growth of α phases on original β grain boundaries, each α platelet maintaining the Burger’s relationship with the parent β matrix [20], as shown in Figure 3e; the second type of GB (Type II) has a smoother appearance in Figure 3d, because α phases precipitating from β matrix have almost the same morphologies and orientations [20]. α platelets may not grow into the entire grain with the increase in the cooling rate; consequently, the narrow-interlaced colony is formed in the middle of the β grain, as seen in Figure 3b,d,f. Moreover, the thickness of α platelets, α colonies, and GB of OFC is thinner than that of FC. It is noticed that the size of α platelets, colonies, and GB decreases with increasing cooling rate.

**Figure 4:**
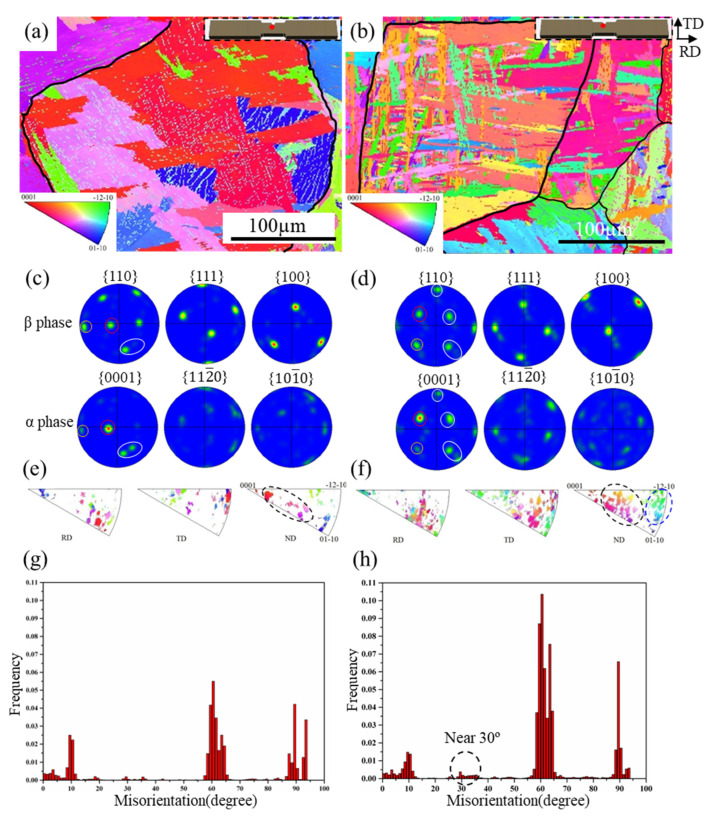
EBSD patterns of (**a**) FC and (**b**) OFC specimens (the insets of upper right show locations on the tensile specimens); pole figures of (**c**) FC and (**d**) OFC specimens; inverse pole figure of α phase in (**e**) FC and (**f**) OFC specimens; and misorientation angle of (**g**) FC and (**h**) OFC specimens.

To further understand the relationship between cooling methods and crystal orientation, EBSD patterns of FC and OFC samples were obtained to analyze the precipitation of α phase, the pole figures, inverse pole figures, and misorientation angle after heat treatment, shown in Figure 4. The Widmanstatten structure is observed in Figure 4a,b. The precipitation of lamellar α phases obeys the BOR of ${\{0001\}}_{\alpha }//{\{110\}}_{\beta }$, ${\langle 11\bar{2}0\rangle }_{\alpha }//{\langle 111\rangle }_{\beta }$ (in Figure 4c,d), forming three major facets: a broad facet (${\left(1\bar{1}00\right)}_{\alpha }//{\left(11\bar{2}\right)}_{\beta }$, ${\langle 11\bar{2}0\rangle }_{\alpha }//{\langle 111\rangle }_{\beta }$), a side facet (${\left(0001\right)}_{\alpha }//{\left(1\bar{1}0\right)}_{\beta }$, ${\langle 11\bar{2}0\rangle }_{\alpha }//{\langle 111\rangle }_{\beta }$), and an edge facet on the interface between the β matrix (growth orientation is ${\left[3\bar{3}5\right]}_{\beta }$). As the broad facet and side facet of α phase obey BOR or near BOR, α phase can only grow on the broad facet through the T-L-K mechanism. α phase is expected to grow quickly along the edge facet due to the high interfacial energy (incoherent interface) of certain atomic planes [21]. Thus, lamellar α phase is formed within the original β grain. However, the orientation of α phase becomes disorderly with an increasing cooling rate, as seen from Figure 4f (mark as dashed blue line). Only five misorientation angles were produced between different α variants: 10°/<0001>, 60°/$\langle 11\bar{2}0\rangle$, 60.83°/$\langle \bar{1.377},\bar{1},2.377,0.359\rangle$, 63.26°/$\langle \bar{10},5,5,\bar{3}\rangle$, and 90°$\langle 1,\bar{2.38},1.38,0\rangle$, with the ratio of 1:2:3:2:2, while the orientation relationship disappeared in both FC and OFC, theoretically due to variant selection (in Figure 4g,h). Moreover, there is an increased frequency of the misorientation angle of 30° in the sample of OFC (in Figure 4h), which indicates that an increasing cooling rate may increase the formation frequency of α variants. Consequently, the orientations of α platelets at a low cooling rate (FC) are more ordered than that at a relatively fast cooling rate (OFC), which can be ascribed to the presence of a large number of nucleation sites, crystal nuclei, and fast growth rate at high cooling rate [22,23].

### 3.2. Mechanical Properties

The mechanical properties of TC21 alloy under different cooling methods are listed in Table 2. The yield strength (YS) increases from 881.68 to 1006.9 MPa, and ultimate tensile strength (UTS) increases from 986.8 to 1114.8 MP, with the increase in cooling rate (from FC to WQ). In particular, the UTS of the FC sample is the lowest (986.8 MPa) but the ductility is the highest (14.6%), and the opposite trend is found for the WQ sample. However, there is an excellent balance between strength and ductility in the sample treated at OFC: the YS and UTS are 928.3 MPa and 1073.48 MPa, respectively, with a moderate elongation of 8.8%. The formation of a thinner α platelet during rapid cooling process and the decrease in microstructure parameters (in Table 1) increase the grain boundaries or phase interfaces of unit volume, which is beneficial to restrict the dislocation or slip at the interface and improve the strength. On the contrary, a decrease in microstructure parameters reduces the slip distance of the dislocation, resulting in a decrease in ductility.

### 3.3. In Situtensile Deformation

**Figure 5:**
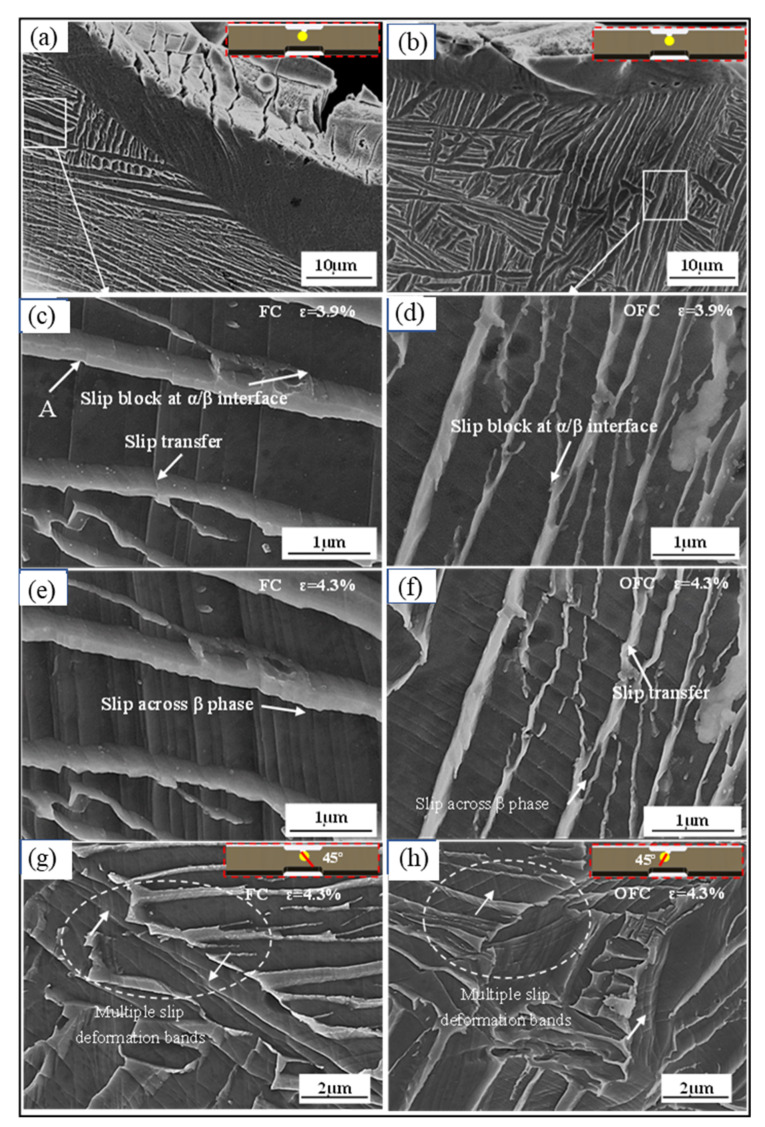
Slip morphologies at ε = 0%, ε = 3.9%, and ε = 4.3% of FC (**a**,**c**,**e**,**g**) and OFC (**b**,**d**,**f**,**h**). The insets show locations on the tensile specimens.

To further reveal the deformation mechanism of the samples obtained at different cooling methods, in situ tensile tests of the FC and OFC samples were performed to observe the deformation process. Naydenkin claimed that the deformation and fracture of the specimens localized in slip deformation bands [24], therefore, it is necessary to observe the morphology of slip deformation bands. Figure 5 shows the slip deformation band morphology of FC and OFC specimens at ε = 3.9% and 4.3%. Most of the slip bands are straight, though some are curved (in Figure 5g,h). At ε = 3.9%, wide slip deformation bands in α platelets and slip deformation steps through α/β interface (arrow A) are shown in Figure 5c,d. However, some slip deformation bands have been blocked by α/β interfaces. The density of the slip bands increases with increasing strain. When the strain reaches 4.3% (in Figure 5e,f), the high-density and narrow slip bands are observed in α platelets and slip bands transfer to β phases. In this case, the slip deformation bands, which are stopped at α/β interfaces, traverse through the interface and move further. However, apparent deformation traces are not observed in the areas between the slip deformation bands, and large shear displacements are produced, as indicated in Figure 5f. In addition, multiple slip deformation bands appear at a strain of 4.3%, as shown in Figure 5g,h. The deformation bands connect two parallel slip bands (pointed by white arrows), which might initiate at one deformation band and then propagate to the other one. Localization of plastic deformation and the intersection of deformation bands with shear displacements can be observed. The shear displacements at the intersections of deformation bands probably result from the formation sequence of the deformation bands, i.e., the post-formed deformation bands shear the pre-formed deformation bands [25]. These observations indicate that the most coordinated plastic deformation is localized in the slip deformation bands.

**Figure 6:**
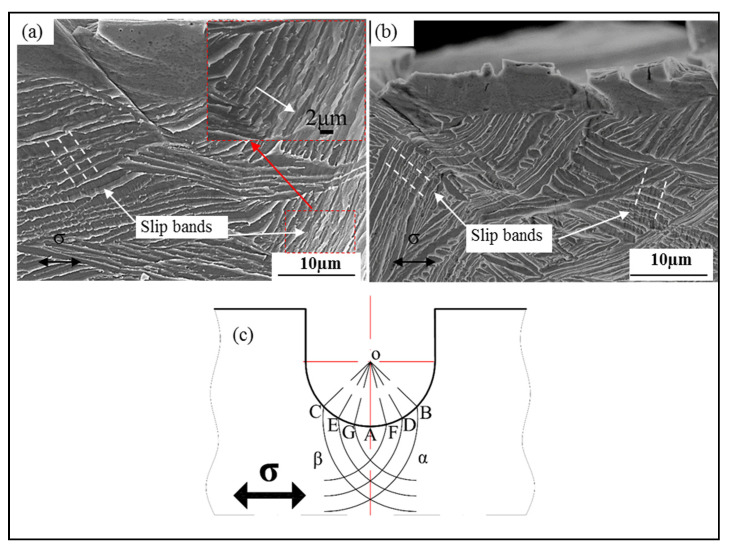
Slip band morphologies during crack initiation and schematic diagram of the slip line field of the circular-notched (**a**) FC and (**b**) OFC specimens. (**c**) The schematic diagram of the slip line field of FC and OFC specimens.

Figure 6a,b show the slip band morphology of FC and OFC at the crack initiation stage, respectively. Slip is the dominant deformation mode both in α platelets and β phase during in situ tensile progress. The slip bands on both sides of the crack are symmetrically distributed and the angle between slipping direction and tensile direction is found to be ~45°. Based on the conventional slip theory, the maximum value of the Schmid factor is 0.5 when the normal direction of the slip surface is 45° from the tensile axis, resulting in minimum critical stress and initiating the slip system. Moreover, the Hencky theorem also pointed out that the direction of the slip line, i.e., the direction of the maximum shear stress, is ~45° to the principal stress axis in a boundary unit [26]. Hui et al. [14] and Zhang et al. [27] also observed that the angle between the tensile slip bands and stress axis is 30–60°. One should note that the given angle is directly related to the actual crystal orientations. Herein, the slip bands (indicated by the arrows in Figure 6a,b) are in two different directions (dashed lines). The formation mechanism of shear bands in two different directions of the circular notch has been analyzed by Hencky theory. Figure 6c presents a schematic diagram of the slip field of a tensile specimen with a circular notch. The stress distribution of the notched specimen is different from the non-notched one. Therefore, the slip in the plastic state proceeds along the direction of the maximum shear stress. The slip lines, presenting the direction of maximum shear stress, are tangent to the maximum shear stress vector, and the angle between the slip lines and principal stress direction is ~45°. As the maximum shear stress at each point is in pairs, the slip lines form two sets of mutually orthogonal lines in the deformed unit, resulting in a slip line field [28]. The irreversible slip occurs on the parallel slip planes inside grains and produces dislocation pileups at the interface, and ultimately leads to crack nucleation.

**Figure 7:**
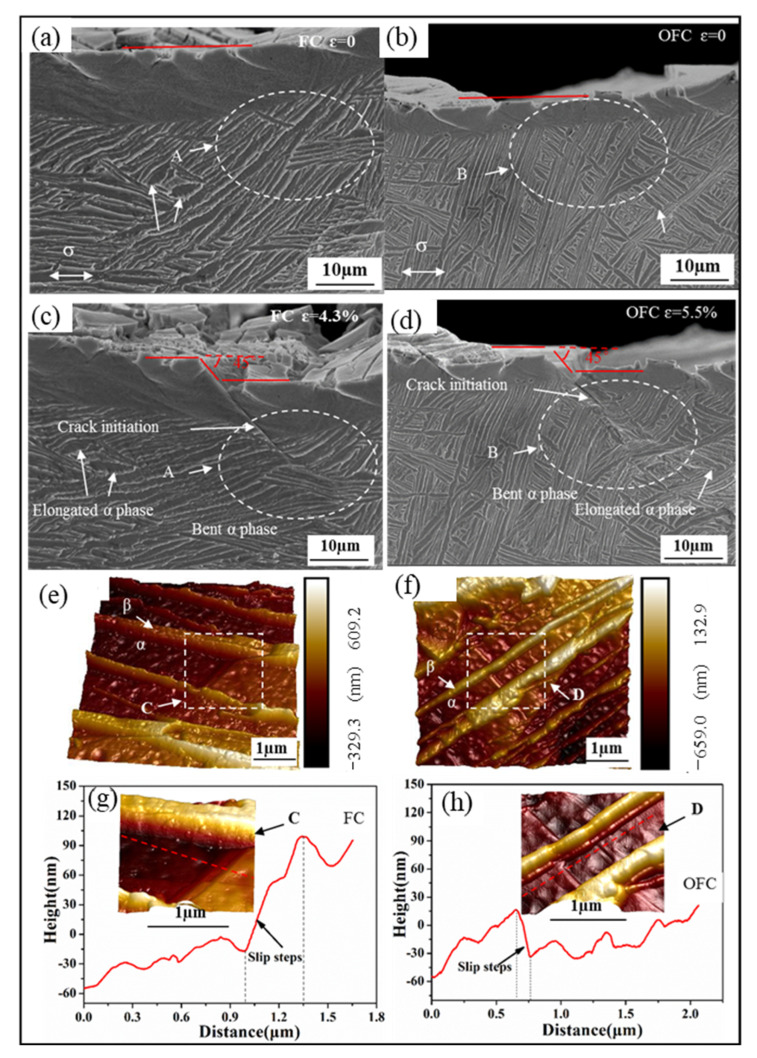
SEM images at different strain and AFM images of crack initiation of FC (**a**,**c**,**e**,**g**) and OFC (**b**,**d**,**f**,**h**).

The SEM images at different strains and AFM images of FC and OFC samples can be seen in Figure 7a,b,d, showing the original (ε = 0) and crack initiation state of FC and OFC samples, respectively. The cracks in the FC and OFC samples initiate at the pre-crack, and the angle between crack direction and tensile axis is found to be 45°. Chan et al. [29] demonstrated that the crack initiates inside the slip bands, which forms on the active slip plane that lies at an angle (e.g., 45°) to the stress axis with a high Schmid factor. Materials with planar slip are known to concentrate slip and form persistent slip bands which eventually lead to the initiation of a crack. As the stress increases and the slip proceeds further, α platelets rotate slightly along the tensile direction. Moreover, local shear deformation occurs due to that shear stress and original α platelets change from straight to curved (marked as A and B in Figure 7c,d, respectively). These curved α platelets break and also can form cracks, which are torn by the shear stress to form the main crack. AFM images (Figure 7e,f,h show that the slip step height of α platelet in FC and OFC samples is 67.5 nm and 49.6 nm, respectively. The magnitude of Burgers vector in Ti alloy is 0.295 nm, i.e., a dislocation slip can result in a slip step of 0.295 nm height [15]. Therefore, the slip step in FC sample (67.5 nm) requires the slip of 229 dislocations whereas only 168 slip dislocations are required in OFC sample. Hence, FC sample possesses superior dislocation tolerance before the crack initiation. Moreover, FC sample presents better ductility because the coarse α platelets provide a longer distance for dislocation slip, which is consistent with the above results in Table 2.

**Figure 8:**
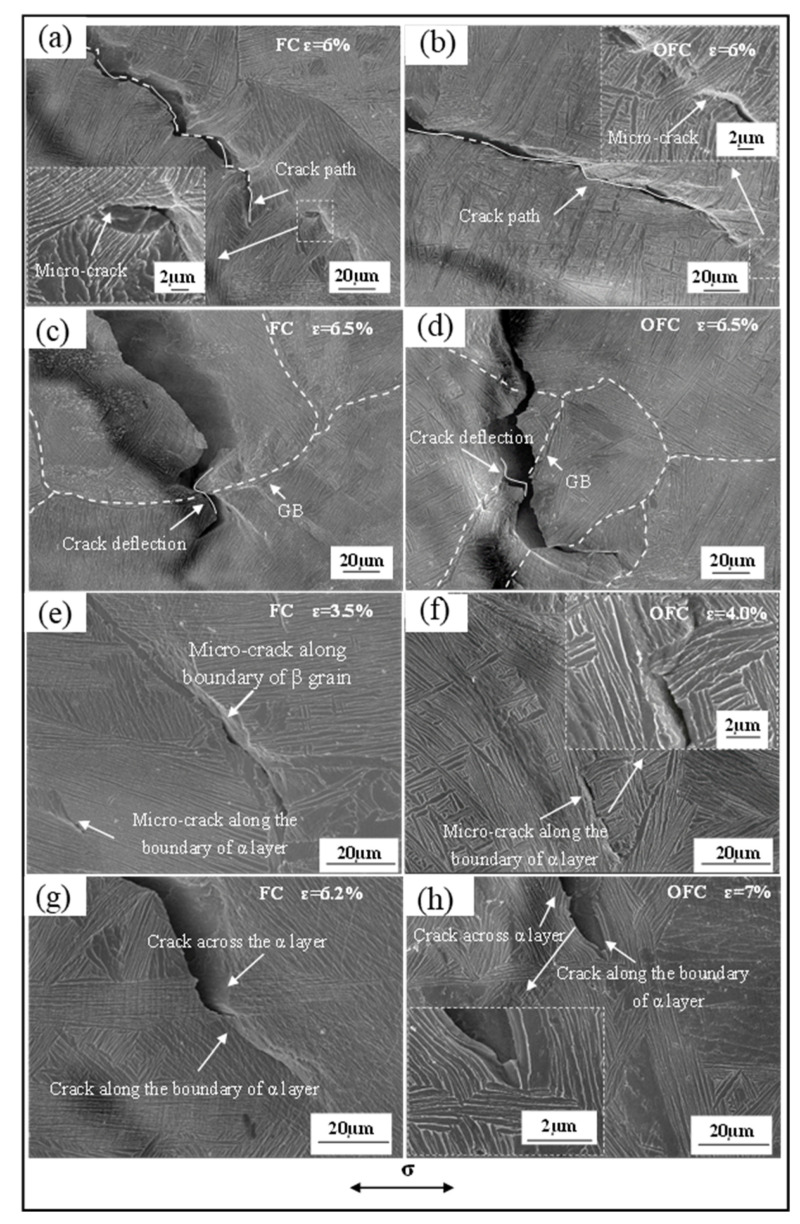
SEM images of crack propagation: (**a**,**c**,**e**,**g**) FC and (**b**,**d**,**f**,**h**) OFC.

Figure 8a,b present SEM images of crack propagation in FC and OFC samples, respectively. The continuous deflection of the crack induced by the crack propagation in α colonies with different orientations can be seen in Figure 8a. However, the macroscopic path deflections have not been observed in OFC samples due to the small size and disordered orientation of α colonies (Figure 8b). Figure 8e,f shows micro-cracks generated at α/β interfaces in FC and OFC samples. Hook et al. [30] have demonstrated that elastic interactive stress at the grain boundaries of polycrystalline material enhanced the local stress concentration. Tan et al. [12] and Holden et al. [31] have argued that the hardness of micron-scale β phase was around three times higher than the micron-scale α phase. Shi et al. [32] have observed that the stiffness of β phase was higher than α phase. Ankem et al. [33] have demonstrated that the final strain of both α and β phases should be equal. Therefore, the stress in α phase increases and the stress in β phase decreases during the deformation process. Moreover, micro-cracks initiate at the stress concentrated grain boundaries or α/β interfaces due to the slip irreversibility. Furthermore, it has been reported that the dislocations tend to nucleate at the interface and then slip in the softer α phase due to stress concentration [10,34]. Figure 8g,h shows two different crack propagation modes: through α colonies and along the α/β interface, depending on the angle between the α/β interface and the crack propagation direction (≤90°). The crack easily propagates through α platelet (or α/β interface) for a large angle (~90°) and along with the α/β interface for a small angle (~0°) between α/β interface and crack propagation direction. Additionally, it is observed that the orientation of α platelet or colony influences the formation of micro-cracks at the interface. All micro-cracks are observed at the α/β interface with an angle (θ, where 0 < θ < π) to the stress axis, as shown in Figure 8e,f.

### 3.4. Fracture Morphology

**Figure 9:**
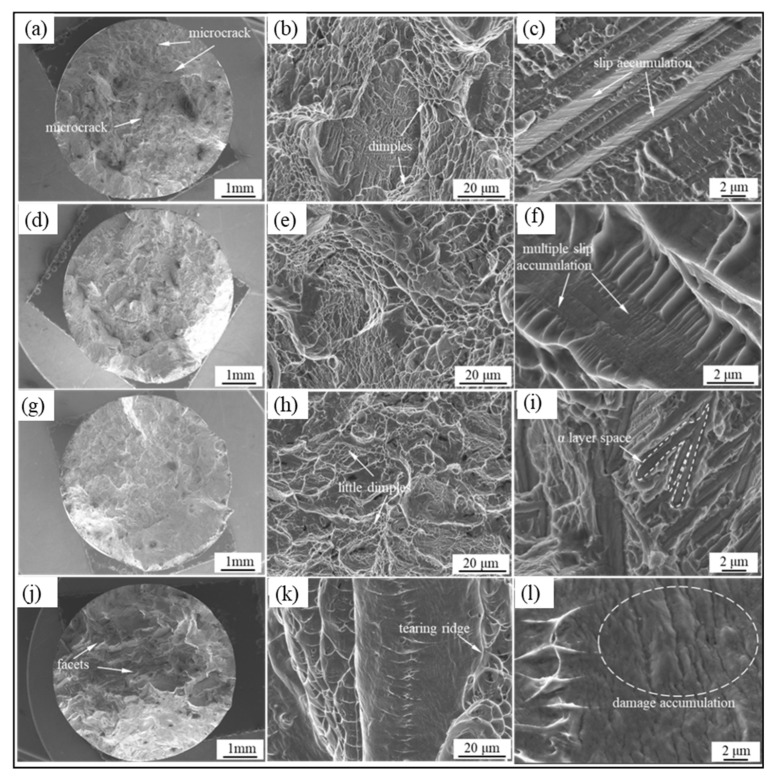
Fracture morphologies of sample: FC (**a**–**c**), OFC (**d**–**f**), AC (**g**–**i**), and WQ (**j**–**l**) at different cooling methods.

Figure 9 shows the fracture surface of the specimens at different cooling methods. The fracture surface becomes flat with an increased cooling rate (Figure 9a,d,g,j), especially many facets occur on the surface of the WQ specimen (Figure 9j). The size and proportion of dimples decrease with the increase in the cooling rate (Figure 9b,e,h,k). In addition, slip accumulation and damage also can be seen on the surfaces (Figure 9c,f,i,l). The above results display a characteristic transition from ductile fracture to quasi-dissociation fracture with an increased cooling rate. Such transition induced by different microstructure features causes the variation in the mechanical properties of the samples.

### 3.5. Discussion

The above results clearly illustrate that the influence of the cooling methods on α platelet microstructure, which leads to the variation of properties in TC21 alloy. In general, the deformation and fracture mechanisms induced by different microstructures are slightly dissimilar, which will also be reflected in the difference in mechanical properties [10].

#### 3.5.1. Key Microstructure Feature Affecting Mechanical Properties

**Figure 10:**
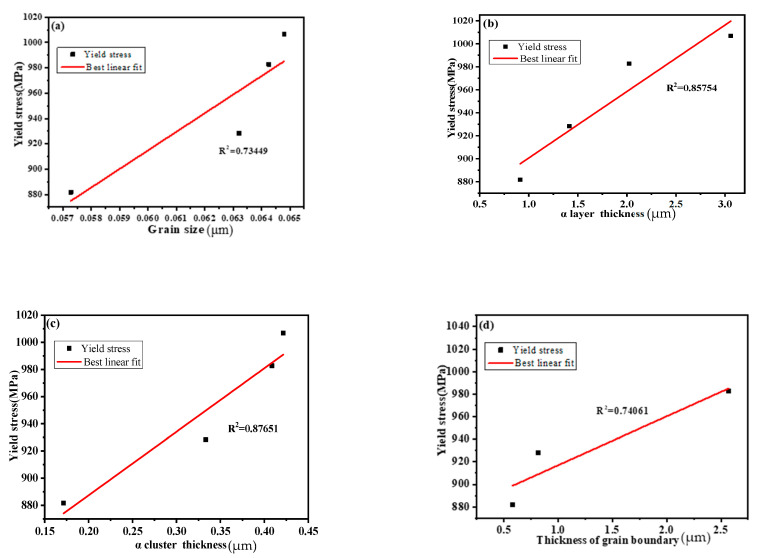

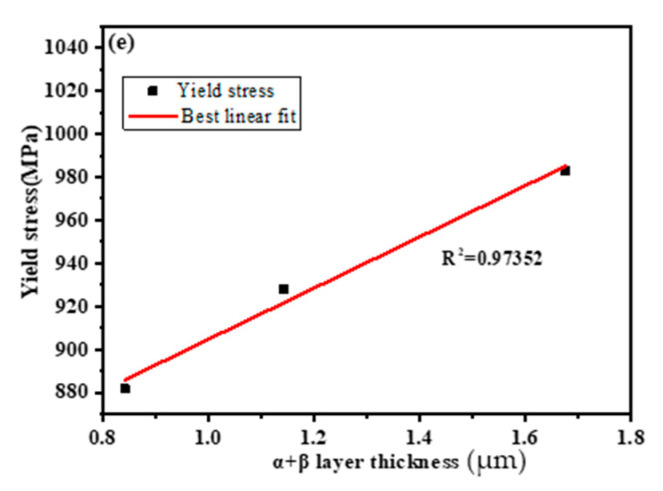
Hall–Petch relationship between yield stress and microstructure parameters including (**a**) Grain size dg, (**b**) α platelet thickness Tα, (**c**) α colony thickness Tc, (**d**) thickness of GB TGB, and (**e**) α+β platelet thickness Tα+β.

As previously mentioned, the cooling methods lead to different α platelet thickness, which plays a crucial role in the mechanical properties of TC21 alloy. The formation of a thin platelet increases the interface that acts as effective dislocation barriers, and thereby improves the strength of the sample. In addition, the lattice defects with high density caused by acicular α martensite (α′, hcp) and α variants also hinder the dislocation slip [21] and increase the strength. However, the decrease in α platelet thickness shortens the dislocation slip distance, and the dislocation pin-up at the interface blocks the slip channel, leading to a decrease in the ductility of the samples. To further reveal that the relationship between the microstructure and the strength, the classic Hall–Petch formula is employed to predict the yield stress of TC21 alloy in the present work, as shown as follows [1]:

(1) $$\sigma_{y}=\sigma_{o}+{\mathrm{kd}}^{-1/2}$$

where σ_y_ is the yield stress and σ_0_ represents the deformation resistance of dislocation slip controlled by the crystal structure and the dislocation density. k is a pin constant and d is the diameter or thickness of microstructure. The relationship between yield stress and the reciprocal square root of microstructure features (including dg, Tα, Tc, TGB, and Tα+β) is presented in Figure 10. As mentioned in Section 3.1 and Section 3.2, the yield stress in the tested samples improves as decreasing dg, Tα, Tc, TGB, and Tα+β. However, the dg, Tα, Tc, and TGB are not the effective control unit of the strength due to the relatively large deviation of the Hall–Petch relationship. One should note that a nearly linear relation (R2 = 0.97) paves between the stress and the reciprocal square root of Tα+β, which reveals that Tα+β is responsible for the stress level of TC21 alloy with platelet microstructure. In addition, the result is a slight collision with the literature [1], in which it is stated that the colony size is an effective control unit of the strength. A possible explanation is that the interface provided by the colony is less due to the lack of consideration for the colony’s component (α or β platelet) with an increased cooling rate. Moreover, the contribution of the β phase to strength is not negligible and the coordination deformation occurs at the lamellar α/β phase. Therefore, the α+β platelets should be related to the strength, and it can promote the strength by providing more effective interfaces to pin dislocations at a fast-cooling rate. In short, the Tα+β is an effective parameter controlling strength in TC21 alloy with platelet microstructure induced by different cooling methods.

#### 3.5.2. Crack Initiation and Propagation

For a bicrystal, the applied stress on α and β phase can be given by Equation (2) under a single stress condition [35]:

(2) $$\sigma_{\alpha }=\left(\sigma A\right)/\left[A_{\alpha }+\left(E_{\beta }A_{\beta }/E_{\alpha }\right)\right]\;\;\;\;\;\;\;\sigma_{\beta }=\left(\sigma A\right)/\left[A_{\beta }+\left(E_{\alpha }A_{\alpha }/E_{\beta }\right)\right]$$

where σ, σ_α_, and σ_β_ represent the total stress and the stress on α and β phase, respectively; A, A_α_, and A_β_ represent the cross-sectional areas of the bicrystal, α phase, and β phase, respectively; E_α_ and E_β_ refer to Young’s modulus of α phase and β phase in the stress direction, respectively. The ratio of σ_α_ to σ_β_ is equal to the ratio of EA to EA, therefore, the phase with a higher modulus of elasticity bears higher stress [31]. Under uniform axial elastic strain, the non-isoaxial bicrystals are not subjected to the same stress. Consequently, the generated interface interactive stress results in elastic incompatibility at the interface, where the crack will initiate (Figure 7 and Figure 8e,f).

**Figure 11:**
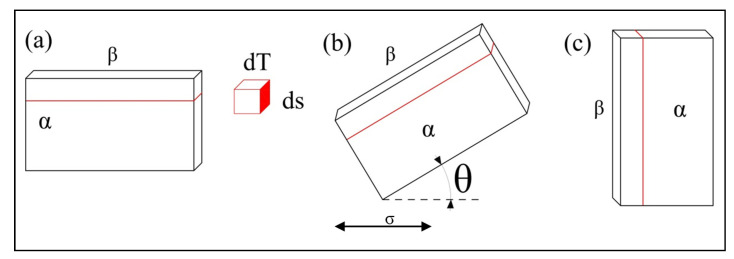
Schematic illustration of (**a**) the interface parallel to the stress axis, (**b**) the interface with an angle (θ) to the stress axis, and (**c**) the interface perpendicular to the stress axis.

Figure 11 presents a schematic illustration of three interfaces in different directions. In a finite-element dT on the interface, when the α/β interface is parallel to the stress axis (Figure 11a), the cross-sectional area in the stress direction becomes ds. When the angle between the interface and stress axis is θ (0 < θ < π, Figure 11b), the cross-sectional area in the stress direction becomes ds/cosθ, which is larger than ds. When the interface is perpendicular to the stress axis (Figure 11c, cosθ = 0), the cross-sectional area in the stress direction becomes infinite. One should note that the cracks are generated due to the formation of micro-voids at the interface [13]. Under the interactive force at the interface, the stressed cross-sectional area increases with the increase in angle (θ) between the interface and stress axis, facilitating the formation of micro-voids at the interface, initiation, and propagation of micro-cracks.

As referenced earlier, crack propagation in two different paths after encountering α colonies in different orientations either pass α colony (along the boundary of α colony or traverse adjacent α colony) or directly traverse the already existing colony. Owing to the large length-to-width ratio of α colony, the energy consumed for bypassing α colony is higher than directly traversing, and the crack prefers to traverse α colony. As α colony is composed of several platelets in the same orientation, once the crack traverses the α colony interface, micro-cracks initially propagate along α platelet interface for a short distance and then propagate through the α platelets. It should be noted that the platelet or colony in the FC sample is thicker than that of the OFC sample. Therefore, the crack completely passes through α colony and produces a macroscopic continuous deflected path (Figure 8c). Moreover, the deflection phenomenon has been observed during crack propagation through grain boundaries [13], as shown in Figure 8c,d. However, the path of crack propagation in OFC is smooth or occasionally deflecting due to the small size and disordered orientation of α colonies (Figure 8b). When crack lip encounters different colonies (Figure 8a), its direction will change and leads to the energy being absorbed. In addition, the change of crack propagating direction or the increased number of cracks can relax the strain-field when the cracks traverse the α/β interface and make the comprehensive properties of FC improve.

**Figure 12:**
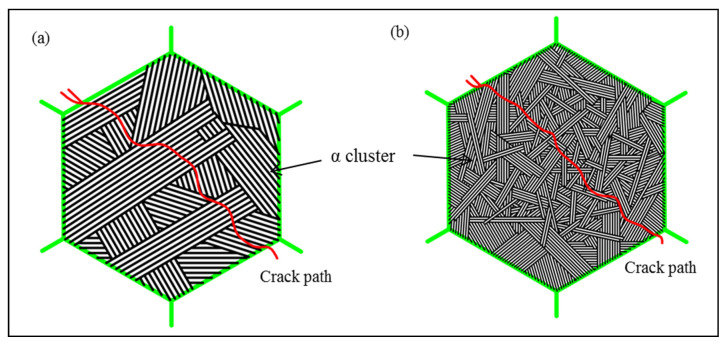
Schematic diagrams of cracks propagation mechanism in FC and OFC (white: α phase, black: β phase, and green: GB). (**a**) FC specimen, (**b**) OFC specimen.

The schematic diagrams of crack propagation mechanisms in the samples with thick α platelets and thin α platelets are illustrated in Figure 12. It is found that the crack in the sample with thick platelets propagates along with the straight slip bands in α colonies. The deflection of the crack path mainly occurs at the interface of the different α colonies, and the degree of deflection depends on the size of α colonies and the crystallographic orientation of the adjacent α colonies. The zigzag crack path reflects the continuous deflection when the crack encounters thick α platelets and wide α colonies in different orientations (Figure 12a). However, a smooth crack path can be seen in the sample with the thin platelets (Figure 12b). The reason for the cracks easily propagating directly through thin α platelets and narrow α colonies with the disordered orientation without deflection based on a minimum energy principle for the inner colonies or the inner platelets in this situation.

In summary, the deformation in thick α platelets and wide α colonies is more difficult than that in thin α platelets and narrow α colonies. Coordinate deformation between wide α colonies consumes more energy compared with narrow α colonies. Moreover, the continuous deflection of the crack occurs in wide α colonies with different orientations, and the larger the deviation angle is, the more energy is required to make the crack propagate.

#### 3.5.3. Deformation and Fracture Mechanism of TC21 Alloy with α Platelets

The slip and shear are the primary deformation modes for the sample with α platelets (Figure 5, Figure 8 and Figure 9). The free slip distance of dislocation depends on the thickness of the platelets. The dislocations start to move along the slip planes with high strain and the encounters of dislocations in the grain generate the jogs, and then lead to vacancies [36]. Moreover, the dislocation pile-up and the coordinative difficulty in deformed α/β interfaces also result in the formation of voids. The aggregation and coarsening of voids will form cracks and cause the final failure of the alloy [34]. It is worth noting that a large number of dimples appear in the surface of FC and OFC specimens (Figure 9b,e) with the minimum size of dimples almost equaling the lamellar α thickness, and the slip accumulation also can be seen in Figure 9c,f, which shows a typical ductile fracture characteristic. However, the proportion of dimples gradually decreases with the increase in cooling rate, and there are many facets appearing on the surface of AC and WQ specimens (Figure 9g,k). Moreover, the coarser vacancies extend, along with the whole α platelet, and form an α platelet space surrounded by tearing edges (Figure 9i). To decrease the energy during crack propagation, two different cleavage planes will form steps which are connected by cleavage facets (perpendicular to main cleavage planes) and the height of cleavage steps decreases with the crack propagation. Therefore, the AC and WQ specimens show a typical cleavage fracture and poor ductility.

## 4. Conclusions

The relationship between lamellar microstructure and the properties of TC21 alloy was systematically investigated, and the tensile deformation process was further revealed by in situ observation. The microstructure, slip, crack initiation, crack propagation, and fracture behavior of specimens with α platelets were analyzed in detail. The main conclusions can be given as follows:

(1) With the increase in the cooling rate, the thickness of the α platelet, α cluster, GB α, and α+β platelet decrease, and the strength of the alloy increases while the plasticity decreases. The thickness of the α+β platelet is the main characteristic factor affecting the mechanical properties.
(2) The zigzag path indicating the continuous deflection of cracks appears when the cracks encounter thick α platelet and wide α colonies with different orientations. However, a smooth crack path can be seen in the specimen with thin α platelets and narrow α colonies, because the cracks can easily propagate through thin α platelets and narrow α colonies with a disordered orientation.
(3) The slip and shear are the primary deformation modes and the ductile and cleavage fractures present the primary fracture mechanism of the specimens with α platelets. However, a large number of large dimples appear on the fractures of the specimen with thick α platelets, indicating a dominating ductile fracture mode.

## Figures and Tables

**Table 1:** Microstructure parameters of TC21 alloy at different cooling methods (μm).

Cooling Methods	Grain Size of β (d_g_)	α Platelet Thickness (T_α_)	α Colony Thickness (T_c_)	Thickness of GB (T_GB_)	α+β Platelet Thickness (T_α+β_)
FC	304.79	1.2	34	3	1.41
OFC	250.31	0.5	9	1.5	0.77
AC	242.19	0.24	5.99	0.15	0.35
WQ	238.15	0.11	5.63	-	-

**Table 2:** Mechanical properties of TC21 alloy under different processes.

Cooling Methods	Yield Strength (MPa)	Ultimate Tensile Strength (MPa)	Elongation A (%)	Percentage Reduction in Area Z (%)	Static Toughness U (MJ/m^3^)
FC	881.9	986.8	10.2	14.6	100.7
OFC	928.3	1073.9	8.8	10.2	94.5
AC	982.8	1100.4	7.1	8.3	78.1
WQ	1006.9	1114.8	6.9	8.2	76.9

## Data Availability

The data presented in this study are available on request from the corresponding author.
